# Is quality affordable?

**Published:** 2008-12

**Authors:** Robert Lindfield, Allen Foster

**Affiliations:** International Centre for Eye Health, London School of Hygiene and Tropical Medicine, Keppel Street, London WC1E 7HT, UK.

**Figure:**
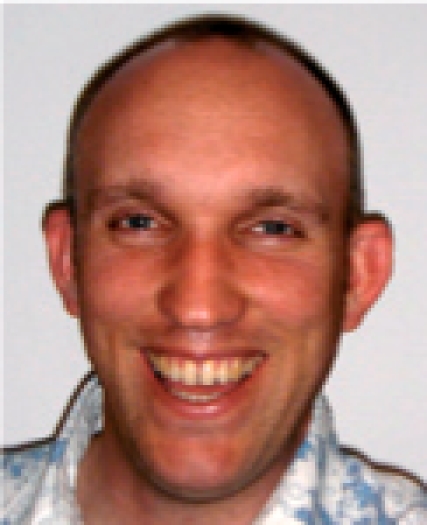


**Figure:**
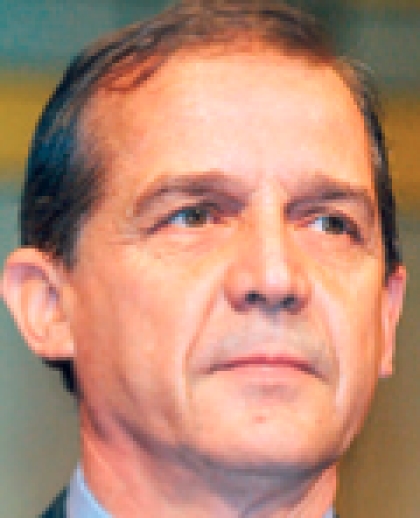


The question “Is quality affordable?” is loaded with dynamite!

Can a person who lives on less than US $1 per day afford a high-quality cataract operation? If the answer is ‘No’, then do we offer that person poor or low-quality services? Do people living in poverty have a ‘right’ to high-quality eye or health care? If the answer is ‘Yes’, then at what price and who should pay? Should we ignore quality and focus on affordability? Or should we provide high-quality services in the hope that someone else will pay?

These are difficult questions, which policy makers, managers, and clinicians must face and try to answer.

## What is quality?

How do we define and measure quality? A simple analogy will highlight the complexity of this issue: if we have a meal, how do we judge its quality? We can measure how many calories, vitamins, etc. the meal contains, or we can decide how satisfied we are with the food, and we may also take the service into account. Our degree of hunger and the price we pay for the meal may influence our level of satisfaction.

**Figure:**
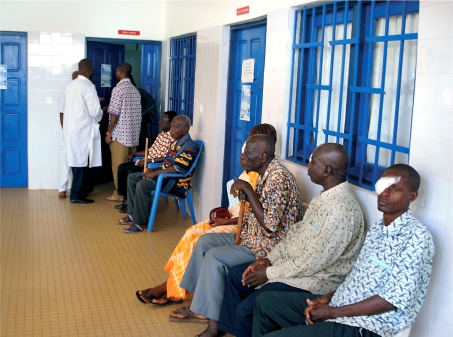
Maintaining clean facilities and clear access for patients contributes to the quality of care. IVORY COAST

The American Medical Association defines the quality of care services as “the degree to which [these] services influence the probability of optimal patient outcomes.”^1^

The World Health Organization offers a more comprehensive defnition^2^ and divides quality in four sections:

1. **Professional performance** (technical quality), including:
  - evidence-based practice
  - clinical audit
  - development of guidelines
  - measures of outcome
2. **Use of resources** (efficiency)
3. **Risk management** (risk of injury or illness associated with the service provided)
4. **Patient satisfaction**

The different aspects of quality have been formulated into a set of six characteristics that any high-quality health programme should display.^3^ As shown in the Box overleaf, such a programme should be: safe, effective, patient-centred, timely, efficient, and equitable.

Quality can vary markedly between organisations. An ophthalmic centre in a high-income country will achieve different outcomes for patients when compared to a low-resource organisation in a low-income country. However, each organisation has a duty to maximise quality within its own resources. Quality is a ‘whole system’ concept: this means that every individual in the organisation, regardless of function or position, should be encouraged to find ways to improve quality.

It is important that we define precisely what quality means to our team or organisation. This will give us clear objectives to improve eye care. Once we have defined quality in our setting, we need to establish ways of measuring and monitoring the different aspects of the quality of eye care. We can objectively measure vision, and whether our intervention restored or preserved it. We can also ask patients about satisfaction, not just with clinical care, but also with the non-clinical aspects of care. Were staff polite? Did they explain procedures? Did patients have to wait a long time?

## Affordability

Affordability depends both on the price of a health intervention and on the financial means of the person or organisation paying for it.

The cost of the intervention or service, and therefore its price, should be kept as low as possible through efficient business practices, e.g. high productivity and no waste (only use what is essential for quality).

Health care can be paid for in several ways: by the government, by the user or family, by another party such as a private company (e.g. health care insurance), or by a nongovernmental development organisation (NGDO). The ability of these organisations or individuals to pay for health care will influence the level of service.

However, if the care needs to be free to some sectors of society, who will subsidise the cost? Sometimes, a family member will pay the fees or the government may provide free health care. The more affluent in society may pay more for services, thereby subsidising services for the poor through a multi-tier paying structure (this is similar to first, business, and economy seats on aeroplanes). Local or international NGDOs may also subsidise costs, but this is less sustainable in the long term.

**Figure:**
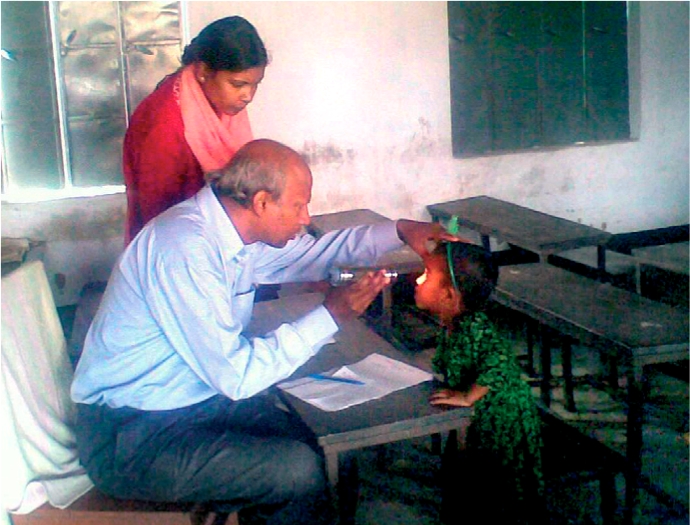
Being attentive to patients' needs is an aspect of quality. BANGLADESH

## Is quality affordable?

There are degrees of quality. An individual organisation or hospital should be able to identify where quality needs to improve and to decide whether such an improvement is affordable.

Affordability of quality is not only or always a question of cost. Cost-effectiveness is important, but so is the best use of resources. Offering services of poor quality is a waste of resources and may prevent uptake of services. Many improvements do not require more money or resources, but may require the team to change the way it works. It is therefore important to consider the situation as a whole.

### Improvements in quality that incur a minimal cost or save money

Some small changes in structure or process can lead to a large improvement in quality. Being aware of the organisation and the way it functions will allow us to identify and address these small changes. Here are two examples:

- A cleaner noticed that the waiting room became very dirty at lunch time and took responsibility for cleaning it before the afternoon clinic. This improved patient satisfaction with the service.
- The nurse in charge of an eye unit conducted an audit into the use of theatre time. She found that if the patient was anaesthetised as the eye doctor was getting ready, then it became possible to carry out ten cataract operations in one session instead of eight. This generated more income for the hospital.

### Improvements in quality that incur a higher cost

We need to decide whether these improvments will be resource-efficient in the long run, as shown by the examples below. If a costly change significantly improves quality, it may be seen as affordable in view of the long-term benefits. Conversely, a procedure may be cost-effective in itself, but it can still represent a misuse of scarce resources.

- An ophthalmologist was keen to move from extracapsular cataract extraction (ECCE) to small incision cataract surgery (SICS). Investigation of the evidence showed a marked difference in surgical outcomes between ECCE and SICS. The cost of moving from ECCE to SICS, whilst large, was felt by the hospital administration to be affordable, because it had the potential to significantly improve clinical outcomes.
- A government hospital in a low-income country wants to set up a corneal eye bank. The ministry of health can only identify a few patients who would benefit from this service and believes that any additional money should rather be spent on cataract surgery, as the need for it is greater. The ministry suggests investing the money in boosting cataract surgical services and reviewing the need for an eye bank at a future date.

### Seeking improvements by focusing on areas of influence

In a resource-poor environment, it can be more difficult to improve quality. For example, if the hospital cannot obtain intraocular lenses (IOLs), then how can it provide a high-quality cataract surgical service? In such instances, we must try to improve quality in the areas where our organisation has influence. We should try to build relationships with an eye hospital that has IOLs, or seek to change the ministry of health's procurement policy through lobbying in the national prevention of blindness committee, or seek support from an external donor.

## Conclusion

We need to make eye care and good vision accessible to everyone regardless of their ability to pay. In order to achieve this, both clinical and non-clinical services need to be of the best possible quality. This requires the involvement of all eye care staff to regularly discuss the quality of care and to identify ways in which practice can be improved with available resources.

**What are the characteristics of a good-quality eye care programme?^3^**

1. **Safe**: avoiding injury to patients
2. **Effective**: based on evidence of effectiveness and avoiding services that have been shown to be ineffective
3. **Patient-centred**: offering care which is responsive and respectful to the patient
4. **Timely**: ensuring that waiting time is minimal, especially for potentially serious disorders
5. **Efficient**: using resources wisely
6. **Equitable**: providing care that does not vary due to personal circumstances or characteristics.

**Improving quality: key messages**

1. **Improvement in quality is part of the day-to-day work** of any eye unit and every eye worker
2. **Both clinical and non-clinical care affect the quality of service**
3. **Quality can be improved in small affordable increments**
4. **Improving quality can save money in the long term** but usually requires some initial investment
5. **Some improvements in quality may not be affordable** at this time and place
6. Each organisation must try to **improve quality within its resource constraints**.
